# Epigenetic Landscape of Kaposi's Sarcoma-Associated Herpesvirus Genome in Classic Kaposi's Sarcoma Tissues

**DOI:** 10.1371/journal.ppat.1006167

**Published:** 2017-01-24

**Authors:** Rui Sun, Xiaohua Tan, Xing Wang, Xiaodong Wang, Lei Yang, Erle S. Robertson, Ke Lan

**Affiliations:** 1 CAS Key Laboratory of Molecular Virology and Immunology, Institut Pasteur of Shanghai, Chinese Academy of Sciences, Shanghai, China; 2 School of Medicine, Hangzhou Normal University, Hangzhou, Zhejiang, China; 3 First Affiliated Hospital of Xinjiang Medical University, Urumqi, Xinjiang, China; 4 Department of Otorhinolaryngology and Tumor Virology Program, Abramson Cancer Center, Perelman School of Medicine at the University of Pennsylvania, Philadelphia, Pennsylvania, United States of America; 5 State Key Laboratory of Virology, College of Life Science, Wuhan University, Wuhan, Hubei, China; National Cancer Institute, UNITED STATES

## Abstract

Kaposi's sarcoma-associated herpesvirus (KSHV) is etiologically related to Kaposi's sarcoma (KS), primary effusion lymphoma (PEL) and multicentric Castleman's disease (MCD). It typically displays two different phases in its life cycle, the default latency and occasional lytic replication. The epigenetic modifications are thought to determine the fate of KSHV infection. Previous studies elegantly depicted epigenetic landscape of latent viral genome in *in vitro* cell culture systems. However, the physiologically relevant scenario in clinical KS tissue samples is unclear. In the present study, we established a protocol of ChIP-Seq for clinical KS tissue samples and mapped out the epigenetic landscape of KSHV genome in classic KS tissues. We examined AcH3 and H3K27me3 histone modifications on KSHV genome, as well as the genome-wide binding sites of latency associated nuclear antigen (LANA). Our results demonstrated that the enriched AcH3 was mainly restricted at latent locus while H3K27me3 was widespread on KSHV genome in classic KS tissues. The epigenetic landscape at the region of vIRF3 gene confirmed its silenced state in KS tissues. Meanwhile, the abundant enrichment of LANA at the terminal repeat (TR) region was also validated in the classic KS tissues, however, different LANA binding sites were observed on the host genome. Furthermore, we verified the histone modifications by ChIP-qPCR and found the dominant repressive H3K27me3 at the promoter region of replication and transcription activator (RTA) in classic KS tissues. Intriguingly, we found that the TR region in classic KS tissues was lacking in AcH3 histone modifications. These data now established the epigenetic landscape of KSHV genome in classic KS tissues, which provides new insights for understanding KSHV epigenetics and pathogenesis.

## Author Summary

Epigenetic modifications are thought to determine the fate of KSHV infection. The epigenetic landscape of KSHV genome in *in vitro* cell culture systems was well studied previously. However, the physiologically relevant scenario in clinical KS tissues is unclear. In this study, we performed ChIP-Seq experiments in classic KS tissues and mapped out the AcH3 and H3K27me3 histone modifications on KSHV genome, as well as the genome-wide LANA binding sites. The results revealed a similar H3K27me3 landscape but distinct AcH3 patterns on the KSHV genome compared to the results from *in vitro* cultured PEL and KSHV infected SLK cells. Intriguingly, there were different LANA binding sites seen on the host genome and a reduced number of AcH3 histone modifications at the TR region of KSHV genome were found. The established epigenetic landscape of KSHV genome in classic KS tissues provides new insights towards our understanding of KSHV epigenetics, which is important for future studies on the mechanism of KSHV infection and pathogenesis.

## Introduction

Kaposi's sarcoma-associated herpesvirus (KSHV) was first identified in Kaposi's sarcoma (KS) biopsies by Chang and Moore in 1994 and has been proven to be the etiological agent of several human cancers including KS, primary effusion lymphoma (PEL) and multicentric Castleman's disease (MCD) [1–3]. KSHV is a double stranded DNA virus with a large genome about 170 Kb, belonging to the gamma herpesvirus subfamily [4, 5]. It typically displays two different phases in its life cycle, the default latency and occasional lytic replication [5]. During latency, the viral genomes persist as episomes with limited latent gene expression in the nucleus of the infected cell and no virion is produced [6, 7]. The latent genes are grouped at one locus in the genome, including ORF73 (latency-associated nuclear antigen, LANA), ORF72 (vCyclin), ORF71/K13 (vFlip), K12 (Kaposin) and a cluster of miRNAs [8, 9]. Under specific conditions such as hypoxia, cell stress and valproic acid or butyrate stimulation, the virus will reactivate from latency with an orchestrated expression of lytic genes, leading to the massive production of mature virions [10–12]. Replication and transcription activator (RTA) encoded by ORF50 is the key switch regulator that controls KSHV reactivation [13, 14]. Adding inhibitors of DNA methyltransferases or histone deacetylases to KSHV infected cells can effectively induce the expression of RTA, which promotes the virus entering lytic replication from latency [11, 15, 16].

Since the epigenetic modifications are thought to determine the fate of KSHV infection, it is important to understand the epigenetic status of viral genome during latency and reactivation [17–20]. Previous studies have elegantly depicted the genome-wide histone modifications on KSHV genome in *in vitro* cell culture systems [21, 22]. It has been demonstrated that activating histone modifications like acetylation of histones (AcH3) are only enriched in several loci while repressive histone modifications like H3K27me3 are widespread across the viral genome, which well explained the expression pattern of viral genes during latency [21, 22]. While most studies are established on the usage of *in vitro* cultured PEL and KSHV-infected endothelial cell lines, the physiologically relevant scenario in clinical KS samples is unclear.

KS is a highly vascular sarcoma on the skin originated from endothelial cells, which is characterized by the infiltrated inflammatory cells and neo-angiogenesis [8, 23]. According to the geographical distribution and clinical outcomes, KS can be classified into four subtypes, which are classic, endemic, iatrogenic and AIDS-related KS. All the subtypes of KS lesions share a common histological characteristic but are substantially different in disease progression [23–26]. The expression pattern of latent genes in KS is not exactly the same as the one in PEL [27, 28]. A previous study has shown that the expression of vIRF3 gene is only detected in PEL samples, which suggests a distinct epigenetic status in KS [28]. Typically, cells derived from KS tissues will loss episomes very quickly during the *in vitro* culture, thus it is difficult to obtain cell lines reflecting the physiologically relevant scenario in KS tissues [29, 30]. Therefore, it is important to directly determine the epigenetic landscape of KSHV genome in KS tissues.

In the present study, we established a protocol of ChIP-Seq for clinical KS samples and directly mapped out the epigenetic landscape of KSHV genome in classic KS tissues which are only associated with KSHV infection and derived from Xinjiang area of China. Specifically, we examined AcH3 and H3K27me3 histone modifications on KSHV genome, as well as the genome-wide LANA binding sites. Our results demonstrated that the enriched AcH3 histone modifications were mainly restricted at latent locus while H3K27me3 histone modifications were widespread on KSHV genome in classic KS tissues. The epigenetic landscape at the region of vIRF3 gene confirmed its silenced gene expression in KS tissues. Meanwhile, the abundant enrichment of LANA at the terminal repeat (TR) region was also validated in the classic KS tissues, however, different LANA binding sites were observed on the host genome. Furthermore, the dominant repressive H3K27me3 histone modifications at RTA promoter region were verified by ChIP-qPCR. Intriguingly, we found that the TR region in classic KS tissues was lacking in AcH3 histone modifications with abundant LANA accumulation. Moreover, the established epigenetic landscape in KS tissues was further confirmed in new cases of classic and AIDS-related KS tissues. By analyzing histone modifications and LANA binding sites in classic KS tissues, our study provides new insights for the understanding of KSHV epigentics.

## Results/Discussion

### Activating AcH3 and repressive H3K27me3 histone modifications on KSHV genome in classic KS tissues

**Fig 1:**
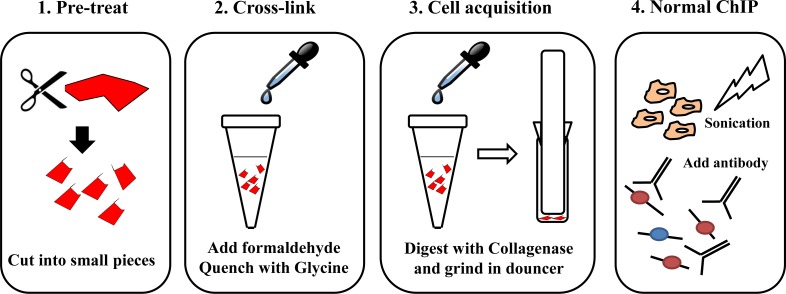
Flowchart of ChIP in KS tissues. Main steps in the protocol include: (1) Pre-treat; (2) Cross-link; (3) Cell acquisition; (4) Normal ChIP procedures.

**Fig 2:**
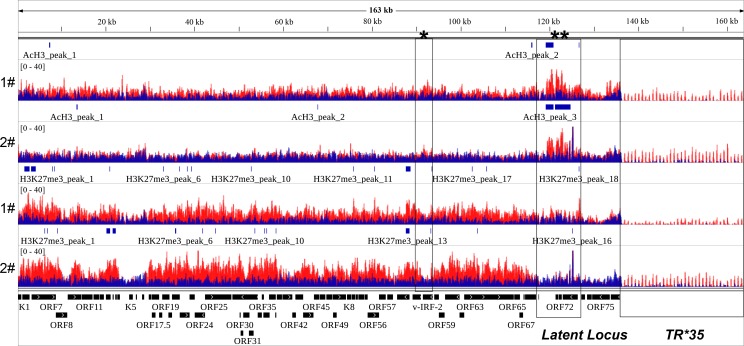
Activating AcH3 and repressive H3K27me3 histone modifications on the KSHV genome in classic KS tissues. The general maps of AcH3 and H3K27 histone modifications on KSHV genome are illustrated. Sequence reads for AcH3, H3K27me3 and Input samples were aligned to the KSHV genome (HQ404500+35TR) and visualized in IGV software. Values shown on the *y* axis represent the relative enrichment of ChIP-Seq signals and has been normalized according to the calculated normalization factors. The epigenetic maps illustrated in the figure contain information from two cases of classic KS tissues. **1#**: the first case. **2#**: the second case. The signals of AcH3 and H3K27 histone modifications were overlaid with the signals of Input (baseline) respectively. **Red**: AcH3 or H3K27. **Blue**: Input. *: the promoter region of vIRF3 gene. **: the latent locus.

**Fig 3:**
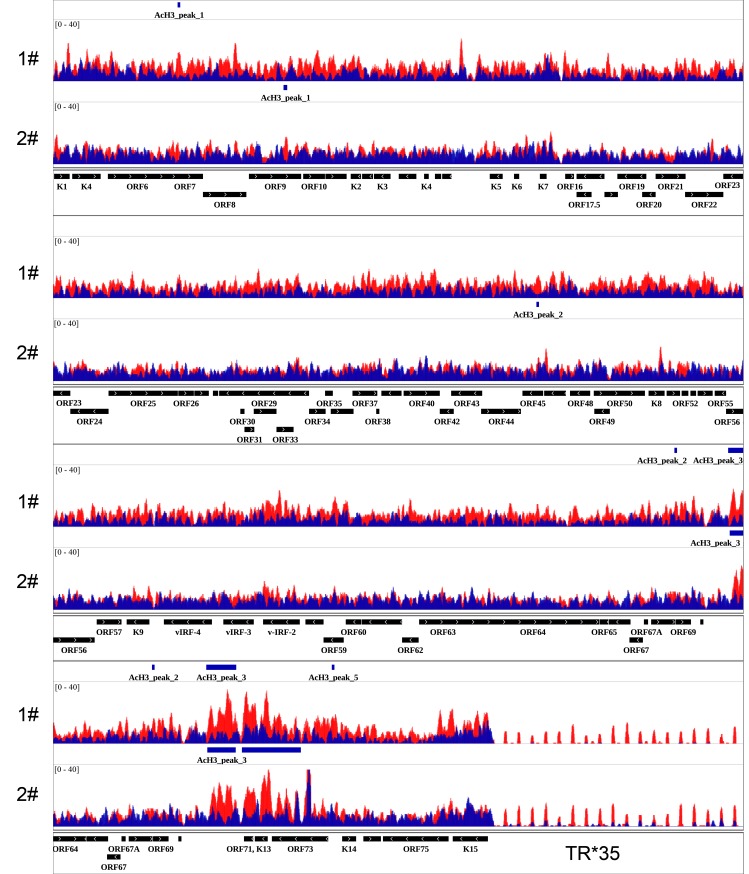
AcH3 histone modification on KSHV genome in classic KS tissues. The enlarged map of AcH3 histone modification on KSHV genome at higher resolution is illustrated. The epigenetic maps illustrated in the figure contain information from two cases of classic KS tissues. **1#**: the first case. **2#**: the second case. The signals of AcH3 histone modification are overlaid with the signals of Input (baseline). **Red**: AcH3. **Blue**: Input.

**Fig 4:**
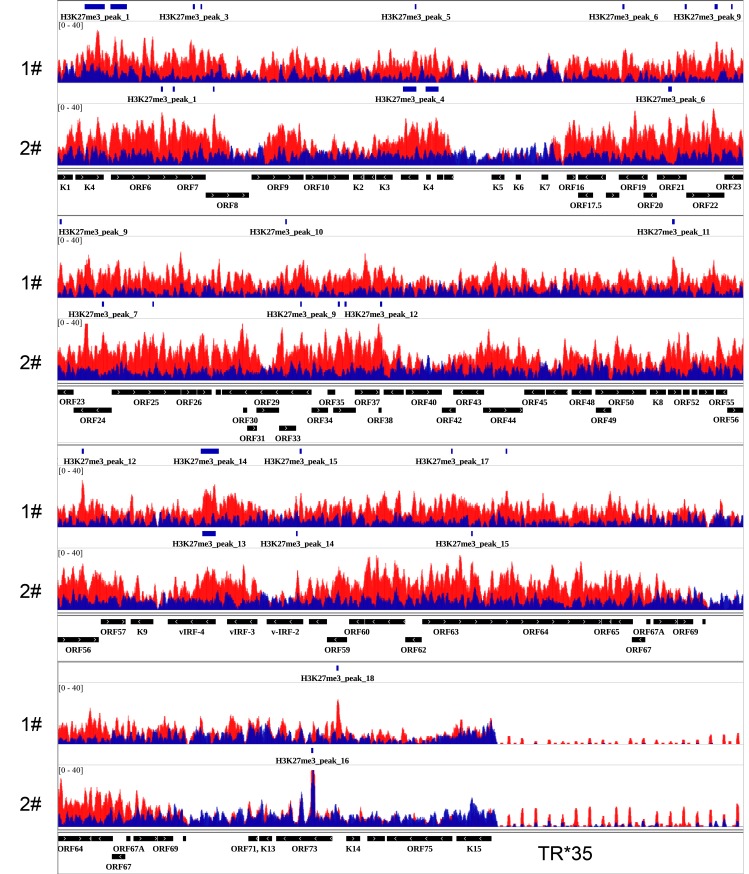
H3K27me3 histone modification on the KSHV genome in classic KS tissues. The enlarged map of H3K27me3 histone modification on KSHV genome at higher resolution is illustrated. The epigenetic maps illustrated in the figure contain information from two cases of classic KS tissues. **1#**: the first case. **2#**: the second case. The signals of H3K27me3 histone modification were overlaid with the signals of Input (baseline). **Red**: H3K27me3. **Blue**: Input.

Previous studies on the epigenetic landscape of KSHV genome using *in vitro* cell culture systems have systemically determined genome-wide distributions of four well-known histone modifications by ChIP-on-ChIP, including AcH3, H3K4me3, H3K27me3 and H3K9me3 [21, 22]. These results supported the predicted activating role of AcH3 and predominant repressive role of H3K27me3 on the KSHV genome. To obtain a physiologically relevant map of the epigenetic landscape of KSHV genome in classic KS tissues, we performed ChIP-Seq experiments on classic KS tissues originated from two different patients. The specific protocol of ChIP in clinical KS tissues was summarized in the Fig 1 and material and methods section. Each experiment was divided into five experimental groups which are input, IgG, LANA, AcH3 and H3K27me3. The purified ChIP product from input, LANA, AcH3 and H3K27me3 groups were subjected to next generation-sequencing. Sequence reads for each sample were aligned to the KSHV genome (HQ404500+35TR) and human genome (Hg19) using Bowtie2 [31]. The results of alignment was presented in Table 1. The overall alignment rate to the KSHV genome was around 0.02%. The relatively high rate in the H3K27me3 group suggested a possible enrichment in KSHV genome. The aligned files were subjected to peak calling and generation of genome-wide maps by using Model-based Analysis of ChIP-Seq (MACS) and Hypergeometric Optimization of Motif EnRichment (Homer) software [32, 33]. The general maps of AcH3 and H3K27 histone modifications on the KSHV genome are illustrated in Fig 2. The enlarged maps are presented in Fig 3 (AcH3) and Fig 4 (H3K27me3). The peak panels illustrated in Figs 2, 3 and 4 demonstrates the most potentially and significantly enriched signals on the KSHV genome. As shown in Fig 2, the enriched AcH3 histone modifications were mainly restricted to the latent locus while H3K27me3 histone modifications were widespread on the KSHV genome. The comparison between AcH3 and H3K27me3 panels showed mutually exclusive signals (high AcH3 level with low H3K27me3 level) on the latent locus and several loci, including the promoter region of vIRF3 gene, regions around ORF8 gene and K5 gene. The coding region of the vIRF3 gene was dominated by the repressive H3K27me3 histone modifications (Figs 3 and 4), which suggests that the vIRF3 gene is silenced but may be easily activated in classic KS tissues. Meanwhile, we also validated that the expression of vIRF3 was restricted at extremely low level in classic KS samples (S1 Fig). This result was in line with previous findings that the vIRF3 expression is not detected in KS tissues by immunohistochemistry analysis [28]. The epigenetic landscape of the KSHV genome in these two cases of classic KS tissues were different from each other in several loci. The first case showed higher level of AcH3 histone modifications on the KSHV genome than the second case in general (Figs 2 and 3), although there was similar trend. To be noted, the promoter and coding regions of the K15 gene were enriched with AcH3 histone modifications only in the first case (Fig 3). In the meantime, we observed a unique peak of H3K27me3 at the promoter region of the LANA gene only in the first case (Fig 4). The higher level of AcH3 and the unique peak of H3K27me3 at the promoter region of the LANA gene in the first case provides supporting evidence for the repressive role of LANA on viral gene expression as previously reported [34–37]. The difference in the overall epigenetic landscape between these two cases indicates different states of KSHV infection in these two patients, which might have clinical relevance to KS progression (S1 File). By comparing with previously published epigenetic maps of the KSHV genome [21, 22], we found that the enriched AcH3 signals at multiple loci (e.g. 10 Kb; 20–30 Kb; 87 Kb) in *in vitro* cell culture systems (BCBL-1 and KSHV infected SLK) were not observed in classic KS tissues while the landscape of H3K27me3 histone modifications in *in vitro* cell culture systems was much similar to the one in KS tissues. It has been reported that KSHV may have different latency programs in different tissues or cell lines and the expression pattern of viral genes could be affected by the cytokines present in the local cellular milieu [27, 38]. The difference in AcH3 histone modification of classic KS tissues might indicate a relatively mild environment with less cytokines for classic KS tissues. Files for the generation of genome-wide maps were provided in the S1 Supporting Information section.

**Table 1:** Overall alignment rate of ChIP-Seq data.

		LANA	Input	AcH3	H3K27me3
Case1	Totol_reads	46573580	55592965	52832914	47653642
	Alignment_Rate _KSHV(%)	0.026	0.010	0.019	0.025
Case2	Totol_reads	37988360	41884249	39835573	37473915
	Alignment_rate_KSHV (%)	0.024	0.016	0.022	0.040

### Genome-wide LANA binding sites in classic KS tissues

**Fig 5:**
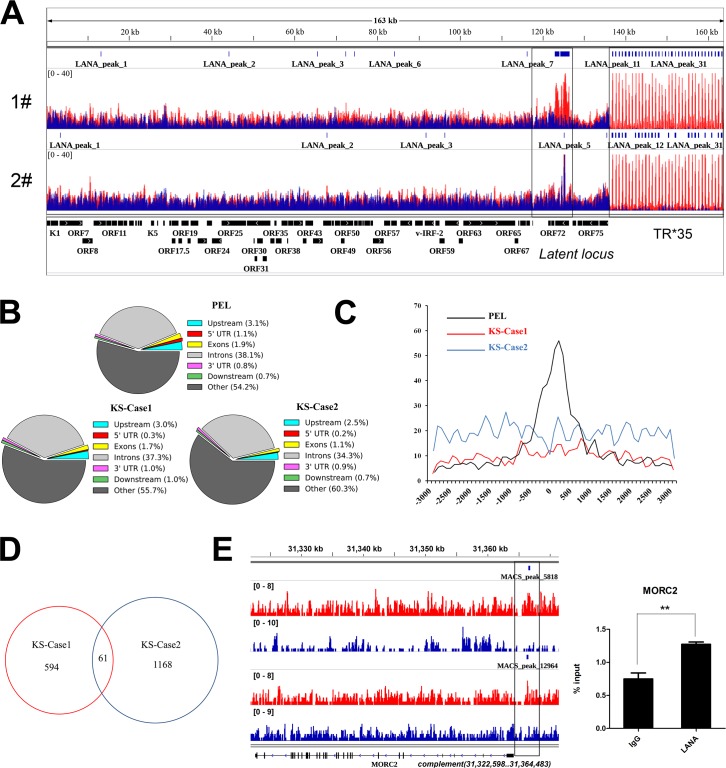
Genome-wide LANA binding sites in classic KS tissues. (A) The general maps of LANA binding sites on KSHV genome are illustrated. Sequence reads for LANA and Input samples were aligned to the KSHV genome (HQ404500+35TR) and visualized in IGV software. Values shown on the *y* axis represent the relative enrichment of ChIP-Seq signals and has been normalized according to the calculated normalization factors. The epigenetic maps illustrated in the figure contain information from two cases of classic KS tissues. **1#**: the first case. **2#**: the second case. The signals of LANA enrichment were overlaid with the signals of Input (baseline) respectively. **Red**: LANA. **Blue**: Input. (B) The distribution of peaks in relation to genes. The identified LANA peaks on the host genome were classified into different groups according to the annotation of peaks by PAVIS. (C) Distances from LANA peaks (-3000 bp to +3000 bp) to TSSs in bins of 100 bp. (D) The number of LANA peaks (-3000 bp to +3000 bp) identified from each case of KS tissues and the number of overlapped peaks. (E) LANA peaks at both the gene body and promoter region of MORC2 gene illustrated by IGV on the GRCh37/hg19. The promoter region of MORC2 gene is illustrated in the box. Bar graph on the right is the result of LANA ChIP–qPCR at the promoter region of MORC2. Data were normalized by the percent input method (signals obtained from ChIP were divided by signals obtained from an input sample) and were presented as mean±SD. The corresponding P value (0.0058) was calculated with Student’s t test.

LANA protein is critical for the maintenance of KSHV episome [39–41]. The genome-wide LANA binding sites in the *in vitro* cell culture systems were well described in several studies [42–47]. Yet its footprint is not known in KS tissues which also shows consistent LANA expression. Therefore, it is important to investigate the behavior of LANA in KS tissues. We designed the experimental group of LANA in the ChIP-Seq experiments. The genome-wide LANA binding sites on the KSHV genome in classic KS tissues are illustrated in Fig 5A. The abundant enrichment of LANA at the terminal repeat (TR) region was validated in both of the two cases of classic KS tissues whereas previously reported the enrichments at the latent locus of LANA was only found in the first case, which further confirmed different states of KSHV infection in these two patients. The panels presented several small peaks across the genome, but was not consistent in these two cases, and was difficult to distinguish from the background noise. Although several weak binding sites of LANA were found on KSHV genome in addition to the TR region and latent locus in the previous studies [42–47], the results in classic KS tissues did not show these peaks. This difference may be a result of the reduced sensitivity of ChIP-Seq for the weaker protein binding sites and the smaller size of classic KS tissues.

In the meantime, we also analyzed LANA binding sites on the host genome. The identified peaks of LANA binding by MACS were subjected to Peak Annotation and Visualization (PAVIS) analysis [48]. The PAVIS result illustrated the relative distribution of LANA peaks in relation to genes (Fig 5B). A dramatic reduction in LANA peaks were annotated at 5' UTR region in KS tissues as compared to PEL cell lines by 3–5 fold. By analyzing the relative distance from peaks to TSS (transcription start site), we found a completely different distribution pattern of LANA binding peaks at the promoter regions in KS tissues as compared to previous studies in PEL (Fig 5C). By cross-comparing the identified LANA peaks in these two KS tissues, we found very few overlapped peaks as shown in Fig 5D and S1 Dataset. The representative overlapped peak was illustrated and validated in Fig 5E. Further comparing with LANA binding sites in PEL, KSHV infected SLK and endothelial cell lines, we found almost no common sites in KS tissues (S1 Dataset). The difference in LANA binding sites on the host genome in KS tissues might suggest a different role for LANA in KSHV pathogenesis, but could not rule out the possibility that the results arose from the cellular heterogeneity in KS tissues.

### Dominant repressive H3K27me3 histone modifications at the RTA promoter region in classic KS tissues

**Fig 6:**
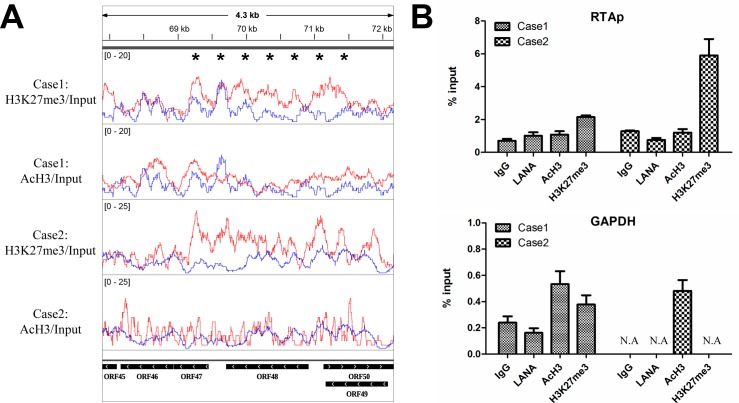
Dominant repressive H3K27me3 histone modifications at the RTA promoter region in classic KS tissues. (A) Illustration of AcH3 and H3K27me3 histone modifications at RTA promoter region (HQ404500: 68000–71000). The signals of AcH3 and H3K27 histone modifications were overlaid with the signals of Input (baseline) respectively. **Red**: AcH3 or H3K27. **Blue**: Input. *: the region of relative strong H3K27me3 enrichment. (B) Results of ChIP-qPCR at the region of RTA promoter and GAPDH gene (control) in classic KS tissues. Samples prepared for ChIP-Seq were divided and a small quantity (1/5) kept for ChIP-qPCR assay before library construction. ChIP–qPCR data were normalized by the percent input method (signals obtained from ChIP were divided by signals obtained from an input sample). Data are presented as mean±SD. **N.A.** represents no amplification.

RTA protein encoded by ORF50 is the key switch regulator that controls KSHV reactivation [13, 14]. The epigenetic status in RTA region may reflect the state of KSHV infection [21, 22]. To validate the epigenetic landscape in the classic KS tissues, we carefully examined and verified the histone modifications at the RTA promoter region by ChIP-qPCR. As shown in Fig 6A, the repressive H3K27me3 histone modifications dominated the RTA promoter region (69–71 Kb) in KS tissues of both patients. The results of ChIP-qPCR also confirmed the enrichment of H3K27me3 at the promoter region of RTA and reflected the differences between these two cases (Fig 6B). Moreover, the enrichment of H3K27me3 could be detected at different regions of RTA promoter in the ChIP-qPCR assay (S2 Fig). The GAPDH region exhibited enrichments of AcH3 and little or no H3K27me3 histone modifications, which was the experimental control for the specificity of LANA, AcH3 and H3K27me3 antibodies (Fig 6B). Previous studies in *in vitro* cell culture systems described a very similar epigenetic landscape at the RTA promoter region as compared to the results in KS tissues [21, 22], implicating that the conclusion from *in vitro* studies about RTA regulation could well support and apply to the *in vivo* scenario.

### Distinct epigenetic landscape at the TR region in classic KS tissues

**Fig 7:**
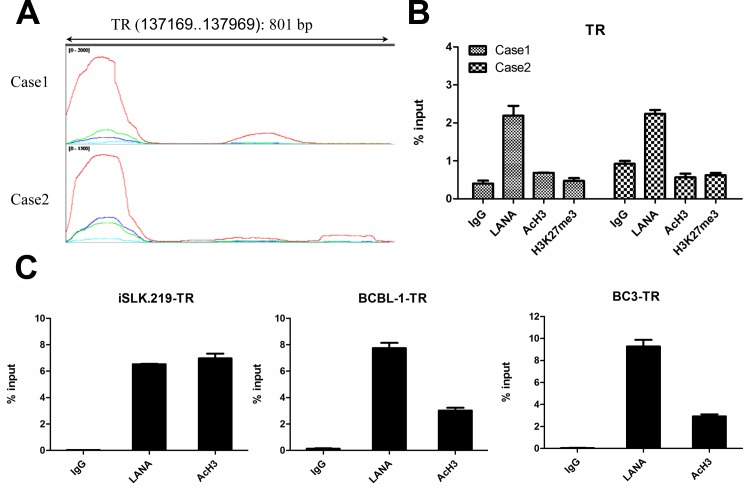
Distinct epigenetic landscape at the TR region in classic KS tissues. (A) Illustration of epigenetic landscape at the TR region (gi|139472801: 137169–137969). Data were presented as line plot in IGV. The signals of AcH3 and H3K27 histone modifications as well as LANA enrichment are overlaid with the signals of Input (baseline). **Red**: LANA. **Grass Green**: AcH3. **Dark Blue**: H3K27me3. **Sky Blue**: Input. (B) Results of ChIP-qPCR at TR region in classic KS tissues. Samples prepared for ChIP-Seq were kept small quantity (1/5) for ChIP-qPCR assay before library construction. ChIP–qPCR data were normalized by the percent input method (signals obtained from ChIP were divided by signals obtained from an input sample). Data are presented as mean±SD. (C) Results of ChIP-qPCR at TR region in *in vitro* cultured cell lines (iSLK.219, BCBL-1 and BC3).

The TR region of KSHV genome consists of highly repeated sequences of 801 bp with multiple copies which can range over 20 Kb [4]. Previous studies have proved abundant AcH3 histone modifications with LANA accumulation at the TR region in *in vitro* cell culture systems [21, 22, 49]. However, we found that the TR region in classic KS tissues was lacking in AcH3 histone modifications with abundant LANA accumulation (Figs 3 and 5A). To analyze the epigenetic landscape at the TR region without consideration of sequence repetition, we re-aligned the sequence reads for each sample to the TR sequence using Bowtie2. The reanalyzed epigenetic landscape at the TR region did not change as illustrated in Fig 7A. To verify the results of the ChIP-Seq, we examined the TR region with the same samples by ChIP-qPCR. As shown in Fig 7B, the enrichment of LANA binding and absence of AcH3 histone modifications were confirmed by ChIP-qPCR. To validate the distinct results in KS tissues, we performed the ChIP experiments in Doxycycline inducible recombinant KSHV.219 harboring SLK (iSLK.219) and body-cavity-based lymphoma (BCBL-1 and BC3) cell lines using the same protocol. The results were shown in Fig 7C. Very strongly enriched signals of LANA binding and AcH3 histone modifications were observed at the TR region as previously reported. The hyperacetylation of histone H3 at TR region was presumably thought to be involved in the assembly of DNA replication factors, yet the significance remained unknown [49]. TR region contains the latent replication origin of KSHV genome, thus hypoacetylation of histone H3 at the TR region might affect the latent replication of KSHV genome, hampering the maintenance of KSHV episomes [40, 50, 51]. This needs further investigation to determine whether losing episomes during the process of *in vitro* culture of cells derived from KS tissues was related to the absence of AcH3 histone modifications at TR region. The hyperacetylation of histone H3 can introduce a loosened chromatin structure at TR region, which may facilitate a poised chromatin structure at the long unique region for the topological speculation. However, the hypoacetylation of histone H3 at the TR region in classic KS tissues was shown to correlate a silenced state of viral genome, thus the acetylation of histone at the TR region may not be related to KSHV gene expression according to the results.

Since KS tumor cells are originated from endothelial cells, we also examined histone modifications and LANA binding sites of KSHV genome in KSHV infected lymphatic endothelial cells (LEC.KSHV). However, the result in LEC.KSHV was also different from the established results in KS tissues. The strong enrichment of LANA binding and AcH3 histone modifications could be observed at the TR region as the same with other *in vitro* cultured cell lines (S3 Fig).

### Validation of histone modifications on the KSHV genome in classic and AIDS-related KS tissues

**Fig 8:**
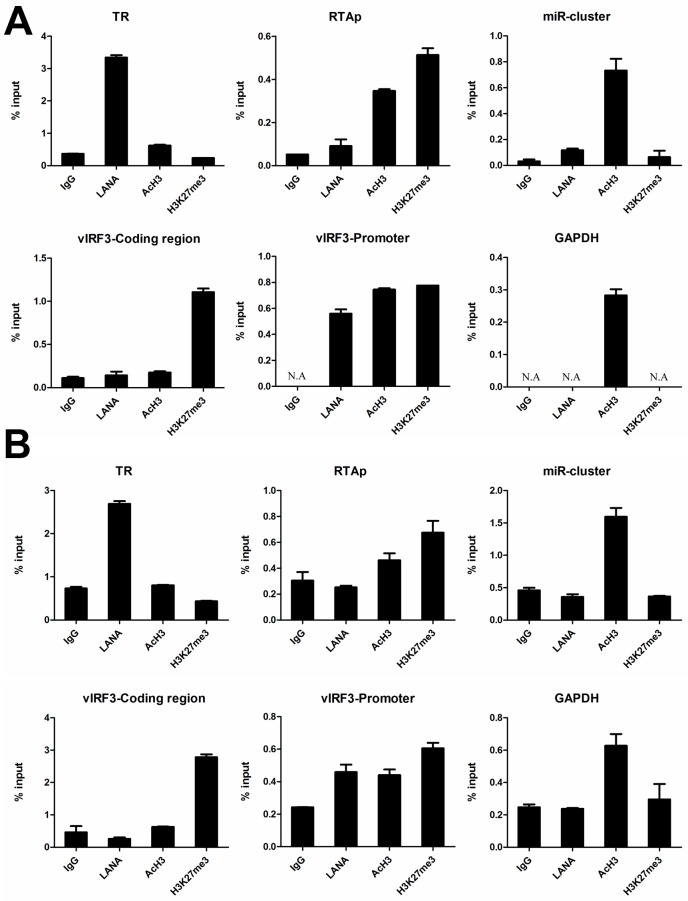
Validation of histone modifications on the KSHV genome in new cases of classic KS tissue. (A) New classic KS case1 and (B) New classic KS case2. Results of ChIP-qPCR at the region of TR, RTA promoter, miR-cluster, vIRF3 and GAPDH gene (control) in classic KS tissue. Samples prepared from new cases of classic KS tissue were used for ChIP-qPCR assay. ChIP–qPCR data were normalized by the percent input method (signals obtained from ChIP were divided by signals obtained from an input sample). Data are presented as mean±SD. **N.A.** represents no amplification.

**Fig 9:**
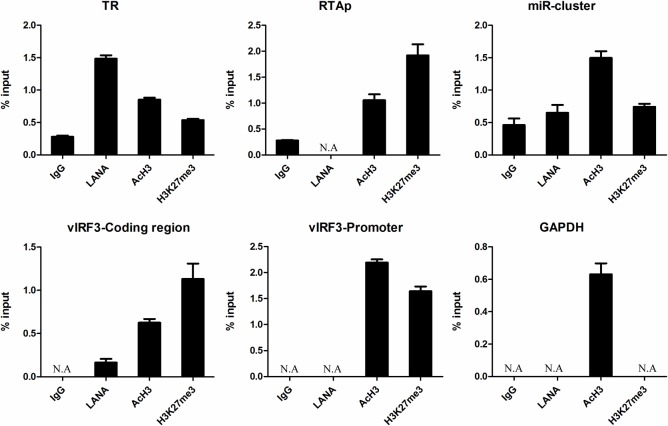
Histone modifications on the KSHV genome in AIDS-related KS tissue. Results of ChIP-qPCR at the region of TR, RTA promoter, miR-cluster, vIRF3 and GAPDH gene (control) in AIDS-related KS tissue. Samples prepared from AIDS-related KS tissue were used for ChIP-qPCR assay. ChIP–qPCR data were normalized by the percent input method (signals obtained from ChIP were divided by signals obtained from an input sample). Data are presented as mean±SD. **N.A.** represents no amplification.

To further confirm the established epigenetic landscape in KS tissues, we examined the epigenetic histone modifications of KSHV genome in new cases of KS tissues (two classic and one AIDS-related). As shown in Fig 8A and 8B, the results of ChIP-qPCR in new cases of classic KS tissue kept good consistency with the previously examined two cases. The established epigenetic landscape at multiple sites were validated, including the TR, RTA promoter, miR-cluster and vIRF3 regions. The general maps of these two cases are illustrated in S4 Fig. Since other subtypes of KS share a common histological characteristic [23], we wondered whether they would have a similar epigenetic landscape as the classic KS tissues. We additionally determined the epigenetic histone modifications of KSHV genome in one case of AIDS-related KS tissue by ChIP-qPCR. Intriguingly, we found the results in AIDS-related KS tissue are similar to the established one in classic KS tissues, but more enrichment of AcH3 histone modifications were observed at the TR and vIRF3 coding region (Fig 9). The difference between classic and AIDS-related samples made us speculate that acetylation of histone H3 at the TR and vIRF3 coding region might correspond to the progression of KS disease. The lesions in classic KS cases are generally localized at the extremities with slow or limited progression while the lesions in AIDS-related KS cases usually spread to the whole body and lead to significant mortality [52–56]. The speculation of a relationship between histone acetylation at the TR and vIRF3 coding region and the progression of KS disease will need a larger subset of cases to be examined to support this hypothesis although the data so far is highly suggestive.

By analyzing histone modifications and LANA binding sites in classic KS tissues, our study established the epigenetic landscape of KSHV genome in clinical KS samples for the first time. The established epigenetic landscape of KSHV genome in classic KS tissues provided direct evidence to support distinct latent programs in KS tissues. A similar epigenetic landscape was observed at the RTA promoter region in KS tissues as compared to the results from *in vitro* cell culture systems, which supported the physiological significance of *in vitro* studies regarding RTA regulation. The distinct AcH3 histone modifications at the TR and vIRF3 coding regions in classic KS tissues provided important clues about the progression of KS disease, which would be a helpful reference for doctors to diagnose clinical patients using epigenetic targeted strategies. We have analyzed the epigenetic landscape of the KSHV genome in classic KS tissues and demonstrated similarities and differences which provide new insights towards understanding KSHV epigenetics, which is important for future studies on the mechanism of KSHV infection and pathogenesis.

## Materials and Methods

### Ethics statement on clinical Kaposi's sarcoma tissues

Experiments in the present study were conducted according to the principles in the Declaration of Helsinki. The usage of clinical Kaposi's sarcoma (KS) tissues was reviewed and ethically approved by the Institutional Ethics Committee of the First Teaching Hospital of Xinjiang Medical University (Urumqi, Xinjiang, China; Study protocol no.20082012). Written informed consent was obtained from all participants, and all samples were anonymized.

### Clinical samples and cell lines

The clinical KS tissues were collected from four patients who had received a pathological diagnosis of KS, including three classic KS and one AIDS-related KS. The patients were of Uygur and Kazak ethnicities from the local region. All samples were collected from Xinjiang province, northwestern China. Details about the patients/specimen were described in the S1 File.

Body-cavity-based lymphoma (BCBL-1) cell line was derived from KSHV positive primary effusion lymphoma patients [57]. BCBL-1 and BC3 was maintained in RPMI 1640 medium (Hyclone) containing 10% fetal bovine serum (FBS) and 5% antibiotics (penicillin and streptomycin, Hyclone). Doxycycline inducible recombinant KSHV.219 harboring SLK (iSLK.219) cell line was established by J. Myoung and D. Ganem [58], and was kindly provided by Fanxiu Zhu (Florida State University). iSLK.219 cell line was cultured in DMEM (Hyclone) supplemented with 10% FBS (Hyclone) and 5% antibiotics (penicillin and streptomycin, Hyclone). Lymphatic endothelial cells (LEC) were purchased from PromoCell (C-12216) and cultured with Endothelial Cell Growth Medium MV2 kit (C-22121, PromoCell).

### ChIP-Seq in clinical Kaposi's sarcoma samples

The collected fresh clinical KS samples (0.1–0.2 g) were stored at -80℃ before usage. The protocol of ChIP-Seq in clinical KS samples is described below:

1 Pre-treat: Cut tissue into small pieces and transfer tissue into a tube with 1 mL PBS.
2 Cross-link: Add formaldehyde to a final concentration of 1.5%. Incubate at room temperature for 20 min. Quench the cross-linking by adding 0.15 mL glycine (1.25 M). Incubate at room temperature for 10 min. Centrifuge at 200 × *g* for 5 min to remove the supernatants and wash the pellet with PBS twice.
3 Cell acquisition: Add Collagenase D or P with Dispase (Roche) to a final concentration of 3 mg/mL. Incubate at 37℃ for 1 h with gentle shaking. Grind tissue in douncer on ice. Use 0.7 μm Falcon filter to get the separated cells. Centrifuge at 300 × *g* for 5 min and wash the pellet with PBS.

(Optional) Enhanced Cross-link: Add formaldehyde to a final concentration of 1.5%. Incubate at room temperature for 3 min. Quench the cross-linking by adding 0.15 mL glycine (1.25 M). Incubate at room temperature for 10 min. Centrifuge at 200 × *g* for 5 min to remove the supernatants and wash the pellet with PBS twice.

4 Normal ChIP procedures: The pellet was lysed with SDS lysis buffer (50 mM HEPES, 1 mM EDTA, 1% SDS, 1mM phenylmethylsulfonyl fluoride [PMSF]) for 10 min on ice. The lysates were subjected to sonication to obtain 200 bp fragments of DNA (Sonics, 2/6 s pulse cycle, amplitude 30–35%), and then centrifuged at 12 000 × g at 4°C for 10 min to obtain the supernatants. The supernatants were diluted 1:10 with RIPA buffer (50 mM Tris–HCl, pH 7.4, 150 mM NaCl, 0.5% Triton X-100, 1 mM PMSF). 10% of the supernatants were kept as input, and the remainder was divided into groups according to the experiment. The aliquots were incubated with pretreated protein A or G beads and corresponding antibody overnight at 4°C. After extensively washing with RIPA buffer, wash buffer (20 mM Tris–HCl, pH 8.0, 1 mM EDTA, 250 mM LiCl, 0.5% NP-40, 1 mM PMSF) and TE buffer (10 mM Tris–HCl, pH 8.0, 1 mM EDTA) (four times each), the beads were resuspended in TE buffer. The resuspended beads were subjected to RNase A and proteinase K digestion, and the crosslinking was reversed at 65°C for 8–10 h. DNA was recycled by DNA purification kit (Tiangen).
5 Library construction and sequencing: The ChIP-seq library was prepared according to the Illumina manual for the preparation of ChIP Sample. The size selection range for the library was 200–400 bp. The established library was validated by Agilent Bioanalyzer and normalized accordingly. The sequencing was performed on the Hiseq 2500 platform.

*Note the pretreated beads should not be blocked with sperm DNA.

Antibodies in the ChIP-Seq experiments: Anti-Trimethyl-histone H3 (Lys27) (H3K27me3) rabbit polyclonal antibody (07–449) was purchased from Merck Millipore. Anti-acetyl-histone H3 (AcH3) rabbit polyclonal antibody (06–599) was purchased from Merck Millipore. Anti-LANA mouse monoclonal antibody produced by 1B5 hybridoma was made in our laboratory (Antigen source for immunization: LANA 900-1162aa) [45].

### Bioinformatics analysis of ChIP-Seq data

The ChIP-Seq data (data quality parameters were described in the S2 File) were aligned to human genome (hg19) and KSHV genome (HQ404500 plus 35 copies of TR [U75699.1]) using Bowtie2 [31]; only one mismatch was allowed. The output files were subjected to peak calling and generation of genome-wide maps with MACS (Model-based Analysis of ChIP-Seq) and Homer2, as described previously [32, 33]. The Input group was used as control. For the analysis of histone modifications with MACS, the parameters were set according to the protocol as followed:—nomodel,—shiftsize = 73. The default P value cutoff for the peak detection was 10^−5.^. For the analysis of histone modifications with Homer2, the adjusted parameters were set as followed: -style = histone, -size = 100 or 150, -minDist = 300. The default P value cutoff for the peak detection was 10^−4^. The final result of identified peaks was generated by the combination of MACS and Homer2 analysis. For the analysis of LANA binding sites with MACS, the parameters were set as previously reported:—nomodel,—shiftsize = 50. The Input group was used as control. The P value cutoff for the peak detection was 10^−3^. Results were visualized by IGV software [59]. Normalization factors were calculated according to the depth of sequencing and formulated as followed: Normalization Factor = Total reads of sample / Total reads of Input. Values on the *y* axis of each panel in IGV software was adjusted according to the calculated normalization factors (S3 File). The peak information was annotated with Peak Analyzer. The distribution of peaks in relation to genes was calculated by PAVIS [48].

### Quantitative real-time PCR (qPCR)

Real-time RT-PCR was performed with a SYBR green Master Mix kit (Toyobo). Reaction mixtures contained 5 μl Master Mix plus Rox, 1 μM each primer, and 4 μl diluted sample. All primers are listed below:

ChIP-TR-F: GGGGGACCCCGGGCAGCGAG
ChIP-TR-R: GGCTCCCCCAAACAGGCTCA
ChIP-RTAp-F: TCCCCTTCTCCACCGTCA
ChIP-RTAp-R: TCCGCAATGTCAGGTTCCAC
ChIP-GAPDH-F: TACTAGCGGTTTTACGGGCG
ChIP-GADPH-R: TCGAACAGGAGGAGCAGAGAGCGA
vIRF3-F (ChIP & RT): GTGTGATCTGCGGACTGTCA
vIRF3-R (ChIP & RT): ACGAGGGGGACCATATCGAA
ChIP-vIRF3p (vIRF2)-F: CTCTGGGACTTGGGAGCAAA
ChIP-vIRF3p (vIRF2)-R: TCTTAACCGCCACCCAATCC
ChIP-miR-cluster-F: TGTGGCACCAGGTTATGGTC
ChIP-miR-cluster-R: GCGTCATGACTAAGGGGGAG
MORC2-ChIP-F: AAGCCCTGTTTGAGTCCCTG
MORC2-ChIP-R: TGCTTTACACTGGGTGCCAT
RTAp-1.6k-F: TCCACAGGACGGCAAATAG
RTAp-1.6k-R: TCCTCATTAGTCGGGACTCG
RTAp-0.4k-F: TCCCAGATCAAAGTCATGTCA
RTAp-0.4k-R: GGCTCAACACCAGACTGAATC

The program set on the 7900HT sequence detection system (Life Technologies) was 95℃ for 5 min, followed by 40 cycles at 95℃ for 15 s, 58℃ for 20 s and 72℃ for 30 s. Melting curve analysis was performed to verify the specificity of the products and each sample was tested in triplicate.

### Accession number

The original data have been submitted to SRA (Sequence Read Archive) in NCBI website. The accession number of this project is SRP081036. KSHV genome: HQ404500. TR: NC_009333, 137169–137969.

## Supporting Information

S1 Fig Expression level of vIRF3 in BCBL-1 cells and classic KS tissues. BCBL-1 cells and classic KS tissues were collected for RNA extraction, and were reverse transcribed to cDNA. The relative quantity was determined by qPCR. Data were normalized against GAPDH (A). To reduce the influence of heterogeneity in KS tissues, the data were also analyzed by normalizing against LANA (B). Data presented as mean±SD.
(TIF)

S2 Fig Enrichment of H3K27me3 at different regions of RTA promoter. Results of ChIP-qPCR at different regions of RTA promoter in classic KS tissue (Case2). Samples prepared for ChIP-Seq were divided and a small quantity (1/5) kept for ChIP-qPCR assay before library construction. ChIP–qPCR data were normalized by the percent input method (signals obtained from ChIP were divided by signals obtained from an input sample). Data are presented as mean±SD. **N.A.** represents no amplification.
(TIF)

S3 Fig Histone modifications on the KSHV genome in KSHV infected LEC. Results of ChIP-qPCR at the region of TR, RTA promoter, miR-cluster and GAPDH gene (control) in KSHV infected lymphatic endothelial cells (LEC). ChIP–qPCR data were normalized by the percent input method (signals obtained from ChIP were divided by signals obtained from an input sample). Data are presented as mean±SD. **N.A.** represents no amplification.
(TIF)

S4 Fig Epigenetic landscape of KSHV genome in new cases of classic KS tissues. Sequence reads for AcH3, H3K27me3, LANA and Input samples were aligned to the KSHV genome (HQ404500+35TR) and visualized in IGV software. Values shown on the *y* axis represent the relative enrichment of ChIP-Seq signals and has been normalized according to the calculated normalization factors. The epigenetic maps illustrated in the figure contain information from two cases of classic KS tissues. The signals of histone modifications and LANA enrichment were overlaid with the signals of Input (baseline) respectively. **Red**: AcH3 or H3K27 or LANA. **Blue**: Input. #: signals at RTA coding region in the LANA group were removed for accidental contamination.
(TIF)

S1 Supporting Information Files for the generation of genome-wide maps. (ZIP)

S1 Dataset Identified LANA peaks in two cases of KS tissues and their overlapped peaks. (XLS)

S1 File Available details about the patients/specimen. (DOCX)

S2 File Data quality parameters. (DOCX)

S3 File Calculated normalization factors. (DOCX)
