# Supplementary figures and images for: Epigenetic Landscape of Kaposi's Sarcoma-Associated Herpesvirus Genome in Classic Kaposi's Sarcoma Tissues
Source: PLoS Pathog. 2017 Jan 24;13(1):e1006167. doi: 10.1371/journal.ppat.1006167 (PMC5291540; doi:10.1371/journal.ppat.1006167)

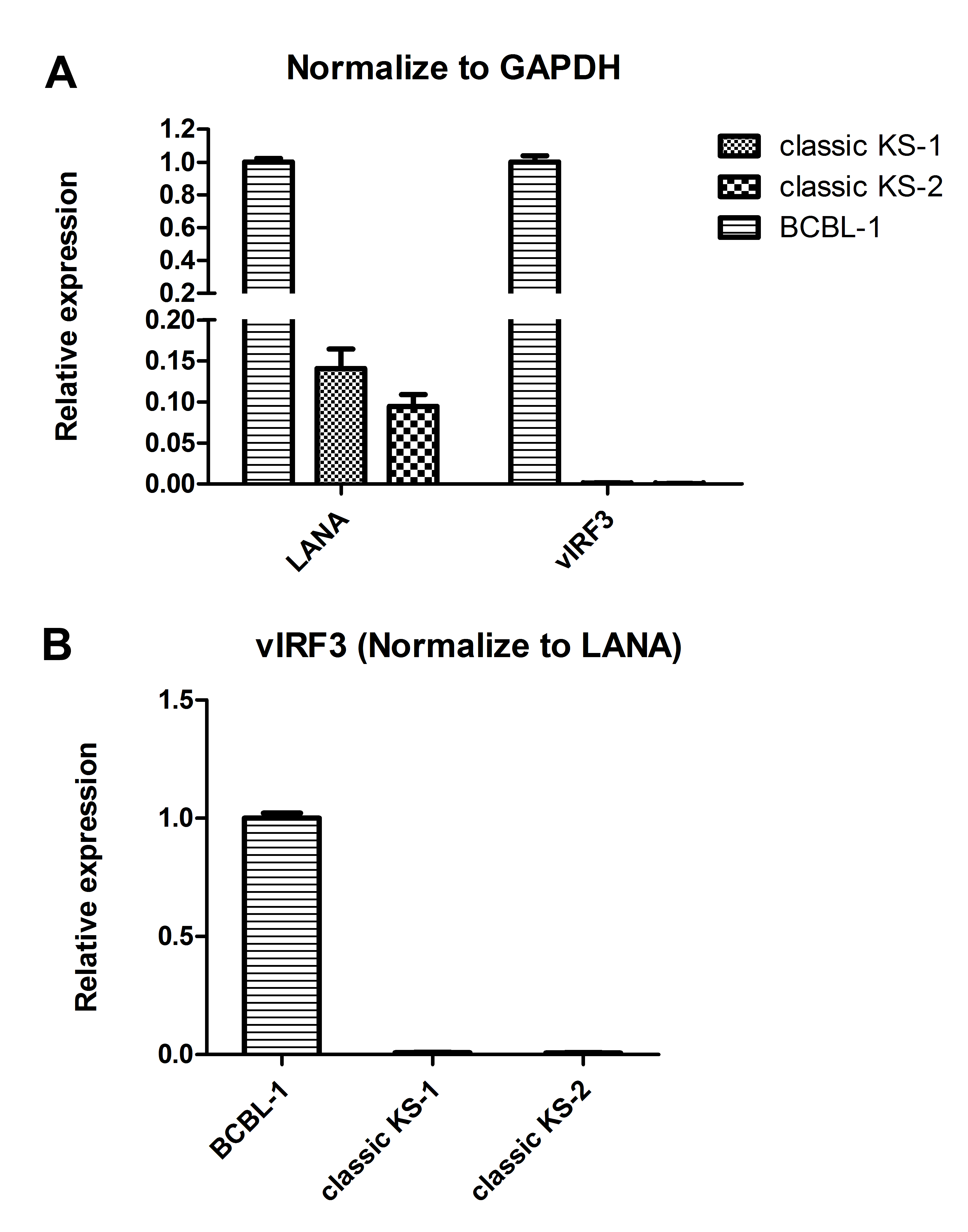

Supplement: S1 Fig — BCBL-1 cells and classic KS tissues were collected for RNA extraction, and were reverse transcribed to cDNA. The relative quantity was determined by qPCR. Data were normalized against GAPDH (A). To reduce the influence of heterogeneity in KS tissues, the data were also analyzed by normalizing against LANA (B). Data presented as mean±SD. (TIF) [file ppat.1006167.s001.tif]

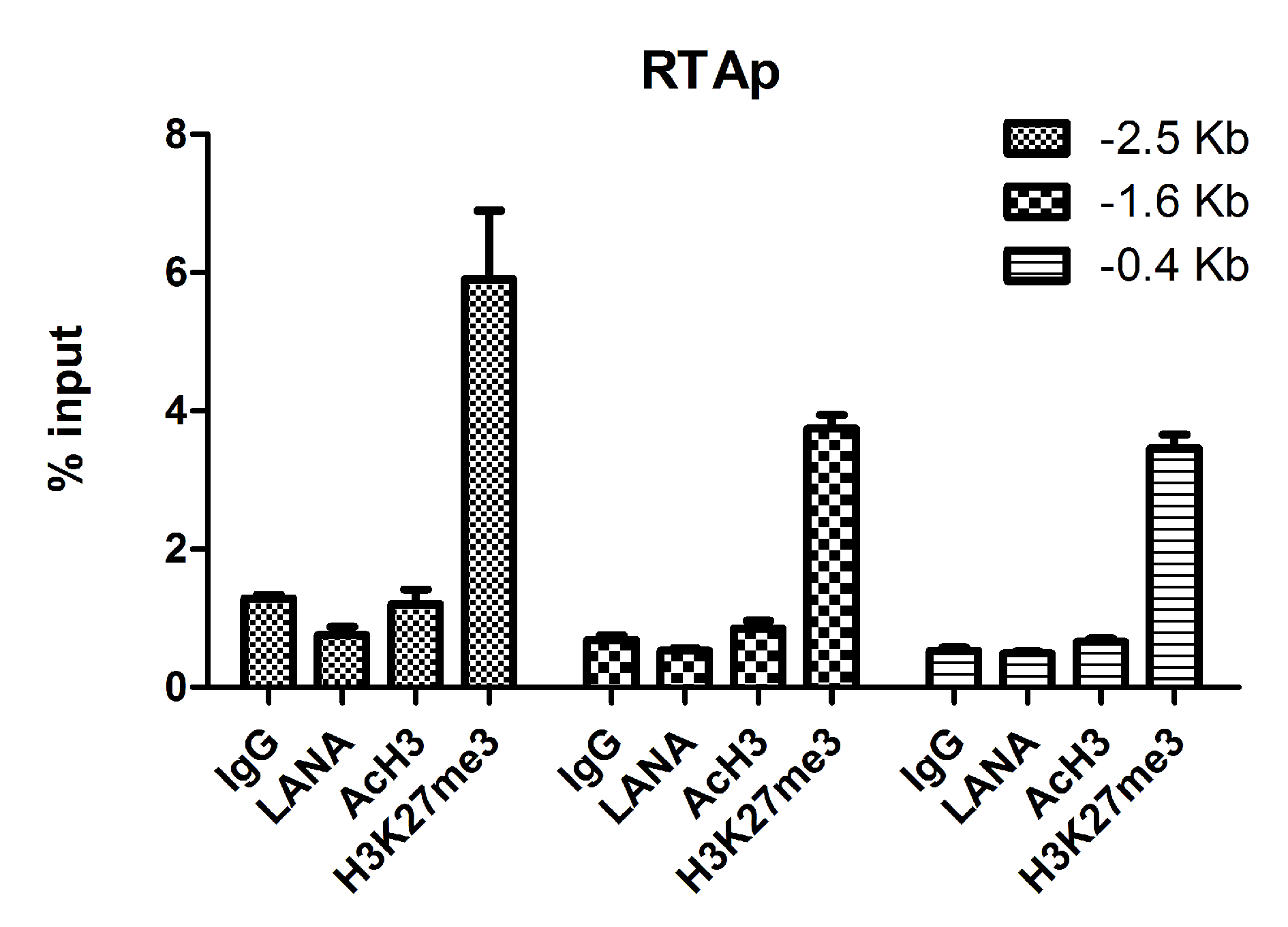

Supplement: S2 Fig — Results of ChIP-qPCR at different regions of RTA promoter in classic KS tissue (Case2). Samples prepared for ChIP-Seq were divided and a small quantity (1/5) kept for ChIP-qPCR assay before library construction. ChIP–qPCR data were normalized by the percent input method (signals obtained from ChIP were divided by signals obtained from an input sample). Data are presented as mean±SD. N.A. represents no amplification. (TIF) [file ppat.1006167.s002.tif]

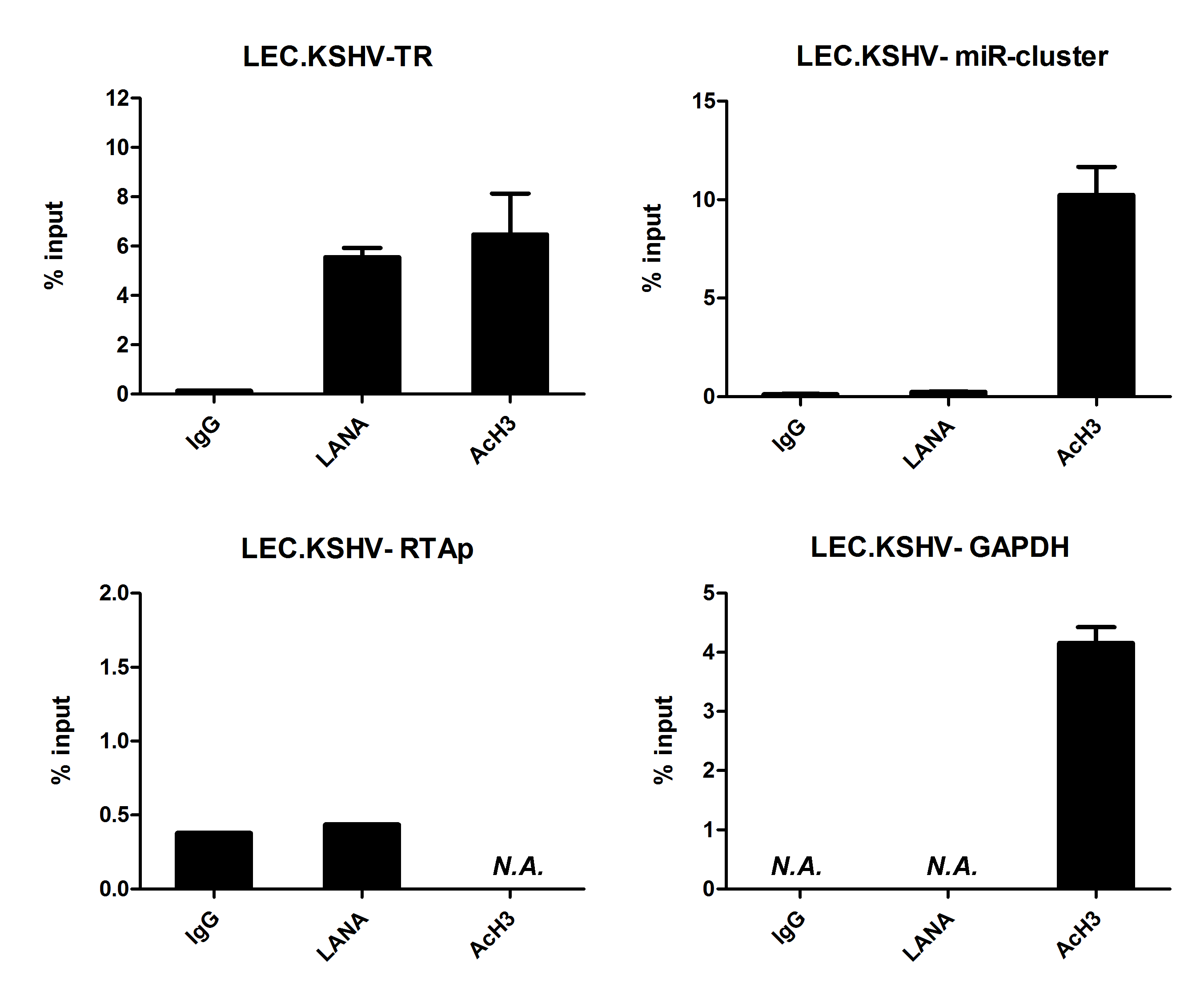

Supplement: S3 Fig — Results of ChIP-qPCR at the region of TR, RTA promoter, miR-cluster and GAPDH gene (control) in KSHV infected lymphatic endothelial cells (LEC). ChIP–qPCR data were normalized by the percent input method (signals obtained from ChIP were divided by signals obtained from an input sample). Data are presented as mean±SD. N.A. represents no amplification. (TIF) [file ppat.1006167.s003.tif]

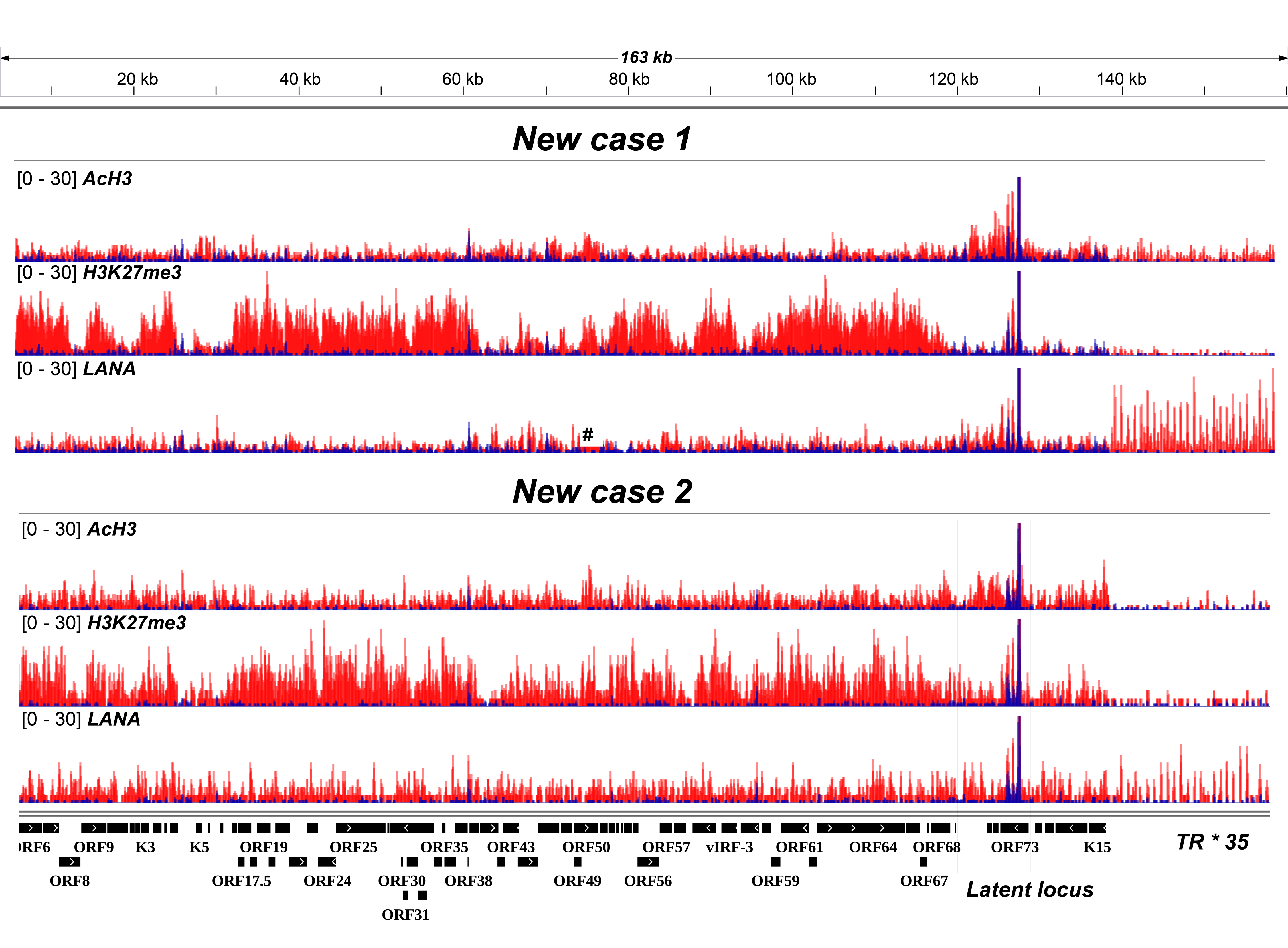

Supplement: S4 Fig — Sequence reads for AcH3, H3K27me3, LANA and Input samples were aligned to the KSHV genome (HQ404500+35TR) and visualized in IGV software. Values shown on the y axis represent the relative enrichment of ChIP-Seq signals and has been normalized according to the calculated normalization factors. The epigenetic maps illustrated in the figure contain information from two cases of classic KS tissues. The signals of histone modifications and LANA enrichment were overlaid with the signals of Input (baseline) respectively. Red: AcH3 or H3K27 or LANA. Blue: Input. #: signals at RTA coding region in the LANA group were removed for accidental contamination. (TIF) [file ppat.1006167.s004.tif]
