# Supplementary material for: Epigenetic Landscape of Kaposi's Sarcoma-Associated Herpesvirus Genome in Classic Kaposi's Sarcoma Tissues
Source: PLoS Pathog. 2017 Jan 24;13(1):e1006167. doi: 10.1371/journal.ppat.1006167 (PMC5291540; doi:10.1371/journal.ppat.1006167)
Supplement: S1 File — (DOCX) [file ppat.1006167.s007.docx]

**S1_File. Available details about the patients/specimen**

| No. | Sex | Age | Ethnic | Address | Course of  Disease (month) | Lesion Location | Lesion Morphology | HIV | Transplant | Note |
| --- | --- | --- | --- | --- | --- | --- | --- | --- | --- | --- |
| Case1  Classic-KS | Male | 25 | Uygur | Shache | 9 | Both lower  extremities | Nodule | (-) | (-) |  |
| Case2  Classic-KS | Male | 53 | Uygur | Artux | 3 | Right upper  extremity | Nodule and patch | (-) | (-) |  |
| New Case1  Classic-KS | - | - | Uygur | - | - | - | - | (-) | (-) | The tissue may be derived from same patient, different lesion tissue |
| New Case2  Classic-KS | Male | 66 | Uygur | Urumqi | 12 | Left lower  extremity | Nodule and patch | (-) | (-) |  |
| AIDS-related  KS case | Male | 31 | Uygur | Kashi | 36 | Right upper  Extremity and body | Plaques | (+) | (-) | Diagnostic report is available**^*^** |

* Diagnostic report summary: Intravenous drug user. Accepted immune therapy for AIDS. TB positive.

CD4: 228 CD8: 1134 CD3:1462

CD4/CD8: 0.19 CD4/CD3: 0.16 CD8/CD3: 0.82.

**Acknowledgement:** We acknowledge the clinical doctors and Dr. Xing Wang, Dr. Xiaohua Tan who provided the valuable clinical information for these samples.
