# Supplementary material for: Epigenetic Landscape of Kaposi's Sarcoma-Associated Herpesvirus Genome in Classic Kaposi's Sarcoma Tissues
Source: PLoS Pathog. 2017 Jan 24;13(1):e1006167. doi: 10.1371/journal.ppat.1006167 (PMC5291540; doi:10.1371/journal.ppat.1006167)
Supplement: S2 File — (DOCX) [file ppat.1006167.s008.docx]

**Data quality parameter (%>=Q30)**

**Case1:**

| **No.** | **Name** | **Reads** | **%>=Q30** |
| --- | --- | --- | --- |
| S15C039 | LANA | 46,573,580 | 94.89 |
| S15C040 | Input | 55,592,965 | 94.25 |
| S15C041 | AcH3 | 52,832,914 | 93.71 |
| S15C042 | H3K27me3 | 47,653,642 | 93.51 |

**Case2:**

| **No.** | **Name** | **Reads** | **%>=Q30** |
| --- | --- | --- | --- |
| O16C001 | LANA | 37,988,360 | 91.72 |
| O16C002 | AcH3 | 39,835,573 | 91.90 |
| O16C003 | H3K27me3 | 37,473,915 | 91.80 |
| O16C004 | Input | 41,884,249 | 92.46 |

**New Case1:**

| **No.** | **Name** | **Reads** | **%>=Q30** |
| --- | --- | --- | --- |
| O16C009 | LANA | 34,797,546 | 91.14 |
| O16C010 | AcH3 | 43,515,069 | 90.25 |
| O16C011 | H3K27me3 | 43,524,045 | 90.80 |
| O16C012 | Input | 52,388,965 | 91.60 |

**New Case2:**

| **No.** | **Name** | **Reads** | **%>=Q30** |
| --- | --- | --- | --- |
| O16C005 | LANA | 35,725,601 | 92.16 |
| O16C006 | AcH3 | 33,213,151 | 91.92 |
| O16C007 | H3K27me3 | 37,598,677 | 92.44 |
| O16C008 | Input | 30,591,756 | 89.18 |
