# Supplementary material for: Epigenetic Landscape of Kaposi's Sarcoma-Associated Herpesvirus Genome in Classic Kaposi's Sarcoma Tissues
Source: PLoS Pathog. 2017 Jan 24;13(1):e1006167. doi: 10.1371/journal.ppat.1006167 (PMC5291540; doi:10.1371/journal.ppat.1006167)
Supplement: S3 File — (DOCX) [file ppat.1006167.s009.docx]

**Calculated normalization factors**

|  | | Input | AcH3 | H3K27me3 | LANA |
| --- | --- | --- | --- | --- | --- |
| Case1 | Totol_reads | 55592965 | 52832914 | 46573580 | 47653642 |
|  | Normalization_Factor | 1 | 0.950353 | 0.837760317 | 0.857188 |
| Case2 | Totol_reads_ | 41884249 | 39835573 | 37988360 | 37473915 |
|  | Normalization_Factor | 1 | 0.951087 | 0.906984389 | 0.894702 |
| New  Case1 | Totol_reads | 52388965 | 43515069 | 43524045 | 34797546 |
|  | Normalization_Factor | 1 | 0.830615 | 0.830786502 | 0.664215 |
| New  Case2* | Totol_reads | 33213151 | 37598677 | 30591756 | 35725601 |
|  | Rates_aligned_to_hg19 | 97.69% | 49.73% | 35.60% | 32.15% |
|  | Reads_aligned_to_hg19 | 32444395 | 18697822 | 10890665 | 11485780 |
|  | Normalization_Factor | 1 | 0.576303597 | 0.335671688 | 0.354014319 |

Normalization Factor = Totol reads of sample / Totol reads of Input.

*Samples were contaminated with sperm DNA (pre-treated beads) in ChIP-seq. Normalization Factor was calculated based on the hypothesis that the rates aligned to human genome is approximate to each other, which has been validated in the other three cases.
